# Correction: Zhou et al. Integrating Chinese Herbs and Western Medicine for New Wound Dressings Through Handheld Electrospinning. *Biomedicines* 2023, *11*, 2146

**DOI:** 10.3390/biomedicines13051033

**Published:** 2025-04-24

**Authors:** Jianfeng Zhou, Liangzhe Wang, Wenjian Gong, Bo Wang, Deng-Guang Yu, Yuanjie Zhu

**Affiliations:** 1 School of Materials & Chemistry, University of Shanghai for Science and Technology, Shanghai 200093, China; 221550217@st.usst.edu.cn (J.Z.); 223353279@st.usst.edu.cn (W.G.); 2 Department of Dermatology, Naval Special Medical Center, Naval Medical University, Shanghai 200052, China; lzwang@hotmail.com (L.W.); m18817365409@163.com (B.W.)

## Error in Figure

In the original publication [1], there was a mistake in Figure 5 as published. The related data were not achieved in the same XRD machine and under the exactly same conditions. Thus, the preparations and XRD tests were repeated. The corrected Figure 5 appears below. The authors state that the scientific conclusions are unaffected. This correction was approved by the Academic Editor. The original publication has also been updated.

## Figures and Tables

**Figure 5:**
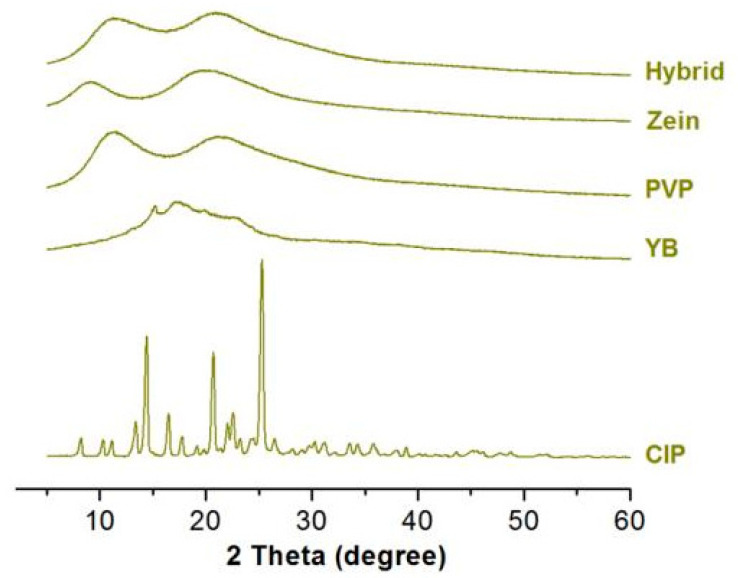
XRD spectra of CIP, YB, PVP, Zein, and Hybrid.
